# Supplementary material for: Social connections at work and mental health during the first wave of the COVID-19 pandemic: Evidence from employees in Germany
Source: PLoS One. 2022 Jun 2;17(6):e0264602. doi: 10.1371/journal.pone.0264602 (PMC9162362; doi:10.1371/journal.pone.0264602)
Supplement: S1 Appendix — (DOCX) [file pone.0264602.s001.docx]

## S2 Appendix. Overview of the RSA methodology.

Since the application of RSA is still rare (Milatz et al., 2015), a short overview of the RSA methodology and our analytical process is given below. The RSA consists of two steps (Humberg et al., 2019). In the first step, the following unconstrained polynomial regression model of second-degree, including the two predictor variables (*X* and *Y*), their squared terms *(*$X^{2}$ and $Y^{2}$), and the interaction of both predictors (*X*Y*) is calculated (Milatz et al., 2015; Nestler et al., 2019):

$$Z=b_{0}+b_{1}X+b_{2}Y+{b_{3}X^{2}+b_{4}Y^{2}+b}_{5}XY+\varepsilon$$

In the second step of the RSA, a three-dimensional graph of the regression model is created. The graph in combination with the regression coefficients and surface parameters is used to interpret the estimated polynomial regression model and to investigate the meaning of the effects more closely. (Humberg et al., 2019; Milatz et al., 2015).

To further guide the interpretation, the checklist of Humberg et al. (2019) was used to identify fit effects (also called congruence effects). To detect fit effects, three features of the response surface are especially important: The ridge of the surface (called First Principal Axis; FPA), the line on the XY plane that contains all congruent predictor combinations *Y = X* (called Line of Congruence; LOC), and the line on the XY plane that contains all incongruent predictor combinations *Y = -X* (called Line of Incongruence; LOIC) (Humberg et al., 2019; Nestler et al., 2019). These features must be considered jointly (Nestler et al., 2019) and have to satisfy at least four conditions to reflect a fit effect (Humberg et al., 2019). To represent a fit effect, the response surface needs to predict the highest outcome for people with congruent predictors (Schönbrodt, 2016). For this, the FPA must not differ significantly from the LOC (Humberg et al., 2019). This is the case when the intercept of the FPA ($p_{10}$) is not significantly different from 0 (condition 1) and the slope of the FPA ($p_{11}$) is not significantly different from 1 (i.e. the confidence interval of $p_{11}$ should include 1; condition 2) (Humberg et al., 2019). Additionally, people with increasingly incongruent predictors need to have significantly lower outcome values (Schönbrodt, 2016). This is the case when the slope of the LOIC at the origin ($a_{3}$) does not significantly differ from 0 (condition 3) and the quadratic term coefficient ($a_{4}$) is significantly negative (condition 4) (Humberg et al., 2019). In sum, the four conditions for fit effects are: (1) $p_{10}$ $\approx$0, (2) $p_{11} \approx$1, (3)$a_{4}$ $<$ 0, and (4) $a_{3}$ $\approx$0 (Humberg et al., 2019). If one of the four conditions is violated, the fit hypothesis must be rejected (Humberg et al., 2019).
