# Supplementary material for: Social connections at work and mental health during the first wave of the COVID-19 pandemic: Evidence from employees in Germany
Source: PLoS One. 2022 Jun 2;17(6):e0264602. doi: 10.1371/journal.pone.0264602 (PMC9162362; doi:10.1371/journal.pone.0264602)
Supplement: S1 Table — (DOCX) [file pone.0264602.s002.docx]

| Construct and Items | | |  | | |  |  |
| --- | --- | --- | --- | --- | --- | --- | --- |
| *Positive Mental Health (9 Items)*  I am often carefree and in good spirits; I enjoy my life; All in all, I am satisfied with my life; In general, I am confident; I manage well to fulfil my needs; I am in good physical and emotional condition; I feel that I am actually well equipped to deal with my life and its difficulties; Much of what I do brings me joy; I am a calm, balanced human being. | | | *α* = .91  *M* = 2.88  *SD* = 0.66 | | |  |  |
| *Mental health disorder (4 Items)*  Little interest or pleasure in doing things; Feeling down, depressed or hopeless; Feeling nervous, anxious or on edge; Not being able to stop or control worrying. | | | *α* = .82  *M* = 1.84  *SD* = 0.64 | | |  |  |
|  | | | Need | Supply | |  |  |
| *Social relationships at work (4 Items)*  The opportunity to talk to others; The opportunity to make friends; being part of a social group; friendly contact with others. | | | *α* = .86  *M* = 3.69  *SD* = 0.89 | | *α* = .86  *M* = 3.15  *SD*=1.08 |  |  |
| *Social support at work (4 Items)*  provide you with support on personal matters; offer you help on personal issues or challenges; offer to listen to a problem you may be having; go out of their way to help you with personal issues. | | | *α* = .95  *M* = 3.25  *SD* = 1.05 | *α* = .92  *M* = 2.81  *SD* =1.13 | |  |  |
|  |  |  |  | | |  |  |

**S1 Table.** Measurement Instruments.

*Note.* *α* = Cronbach’s alpha, *M* = Mean, *SD* = Standard Deviation
