# Supplementary material for: Social connections at work and mental health during the first wave of the COVID-19 pandemic: Evidence from employees in Germany
Source: PLoS One. 2022 Jun 2;17(6):e0264602. doi: 10.1371/journal.pone.0264602 (PMC9162362; doi:10.1371/journal.pone.0264602)
Supplement: S3 Table — (DOCX) [file pone.0264602.s004.docx]

**S4 Table.** Sensitivity analysis two: Re-estimated polynomial regression model using the PHQ-2 to assess mental health disorders.

|  | $b_{0}$ | $b_{1}$ | $b_{2}$ | $b_{3}$ | $b_{4}$ | $b_{5}$ |
| --- | --- | --- | --- | --- | --- | --- |
| b) Social interaction and mental health disorders | 1.82*  [+0.08] | -0.21*  [-0.02] | 0.10*  [+0.02] | 0.00  [0.00] | -0.02  [0.00] | 0.03  [-0.01] |
| d) Social support and mental health disorders | 1.86*  [+0.08] | -0.15*  [-0.01] | 0.14*  [+0.02] | 0.36  [-0.00] | -0.10*  [-0.01] | 0.01  [0.00] |

*Note.* Full polynomial regression model $Z=b_{0}+b_{1}*X+b_{2}*Y+b_{3}*X^{2}+b_{4}*XY+b_{5}*Y^{2}$; *X* = supplies; *Y* = needs; Columns show regression coefficient estimates and coefficient changes in comparison to the original model [in parenthesis]; significance changes are indicated in **bold**; * *p* < .05
